# G9a/GLP-dependent H3K9me2 patterning alters chromatin structure at CpG islands in hematopoietic progenitors

**DOI:** 10.1186/1756-8935-7-23

**Published:** 2014-09-10

**Authors:** Dustin E Schones, Xiaoji Chen, Candi Trac, Ryan Setten, Patrick J Paddison

**Affiliations:** 1 Department of Cancer Biology, Beckman Research Institute, City of Hope, Duarte, CA 91010, USA; 2 Human Biology Division, Fred Hutchinson Cancer Research Center, Seattle, WA 98109, USA

**Keywords:** Chromatin accessibility, CpG island, G9a, GLP, H3K9me2, Hematopoietic progenitor

## Abstract

**Background:**

The formation of chromatin domains is an important step in lineage commitment. In human hematopoietic stem and progenitor cells (HSPCs), G9a/GLP-dependent H3K9me2 chromatin territories form *de novo* during lineage specification and are nucleated at punctate sites during lineage commitment. Here, we examined the patterning of G9a/GLP-dependent H3K9me2 in HSPCs and the consequences for chromatin structure.

**Results:**

We profiled chromatin accessibility across the genome of HSPCs treated with either a small molecule inhibitor of G9a/GLP or DMSO. We observed that chromatin accessibility is dramatically altered at the regions of H3K9me2 nucleation. We have characterized the regions of H3K9me2 nucleation, with our analysis revealing that H3K9me2 is nucleated in HSPCs at CpG islands (CGIs) and CGI-like sequences across the genome. Our analysis furthermore revealed a bias of H3K9me2 nucleation towards regions with low rates of C- > T deamination, which typically lack DNA methylation. Lastly, we examined the interaction of H3K9me2 and DNA methylation and determined that chromatin accessibility changes upon loss of H3K9me2 are dependent on the presence of DNA methylation.

**Conclusions:**

These results indicate that H3K9me2 nucleation is established at specific sequences that have base composition similar to CGIs. Our results furthermore indicate that H3K9me2 nucleation leads to local changes in chromatin accessibility and that H3K9me2 and DNA methylation work synergistically to regulate chromatin accessibility.

## Background

G9a/EHMT2 and GLP/EHMT1 are conserved protein lysine methyltransferases that play key roles in regulating gene expression and chromosome structure during mammalian development through *de novo* mono- and di-methylation of histone H3 lysine 9 (H3K9me1/2), histone marks associated with transcriptional repression [1-5]. During embryogenesis, large G9a/GLP-dependent H3K9me2 chromatin territories arise that have been proposed to reinforce lineage choice by determining higher order chromatin structure [4].

We recently observed that in adult human hematopoietic stem and progenitor cells (HSPCs), H3K9me2 chromatin territories are absent in primitive cells and are formed *de novo* during lineage commitment [6]. In committed HSPCs, G9a/GLP activity nucleates H3K9me2 marks at CpG islands (CGIs) and other genomic sites, and this mark then spreads to form larger domains during differentiation [6]. A recently developed small molecule inhibitor of G9a and GLP, UNC0638, inhibits the methyltransferase activity of both proteins by blocking substrate access to the SET domains [7]. We have shown that treatment of HSPCs with UNC0638 results in a genome-wide loss of H3K9me2, a less dramatic reduction in H3K9me1 and no effect on H3K9me3 or the expression of G9a [6]. These results are consistent with previous studies showing that loss of G9a leads to loss of H3K9me1/me2 [8,9]. We furthermore observed that HSPCs treated with UNC0638, a G9a/GLP small molecular inhibitor, better retain stem cell-like phenotypes and function during *in vitro* expansion and increased expression of lineage-affiliated genes and certain gene clusters, suggestive of changes in regulation of chromatin structure [6].

Primitive hematopoietic stem cells (HSCs) have been hypothesized to have a more “open” chromatin structure that might help maintain a multipotent state by, for example, allowing transcriptional priming of lineage-affiliated genes [10-12]. One possible interpretation from our previous data is that G9a/GLP-H3K9me2 patterning helps restrict chromatin accessibility to reinforce lineage commitment. To investigate this, we examined the consequences of G9a/GLP-dependent H3K9me2 patterning on chromatin structure in HSPCs using FAIRE-seq (Formaldehyde Assisted Isolation of Regulatory Elements Sequencing) [13] to map accessible chromatin in CD34^+^ HSPCs treated with UNC0638 or dimethyl sulfoxide (DMSO) control (see Methods and Additional file 1: Table S1). We furthermore investigated the sequence features of sites of H3K9me2 nucleation. Our results indicate that H3K9me2 is nucleated at CGI-like sites across the genome, with a bias towards regions with low rates of C- > T deamination. Our results further demonstrate that H3K9me2 nucleation is associated with loss of chromatin accessibility and that changes in chromatin accessibility corresponding to loss of H3K9me2 are dependent on the presence of DNA methylation.

## Results and discussion

**Figure 1:**
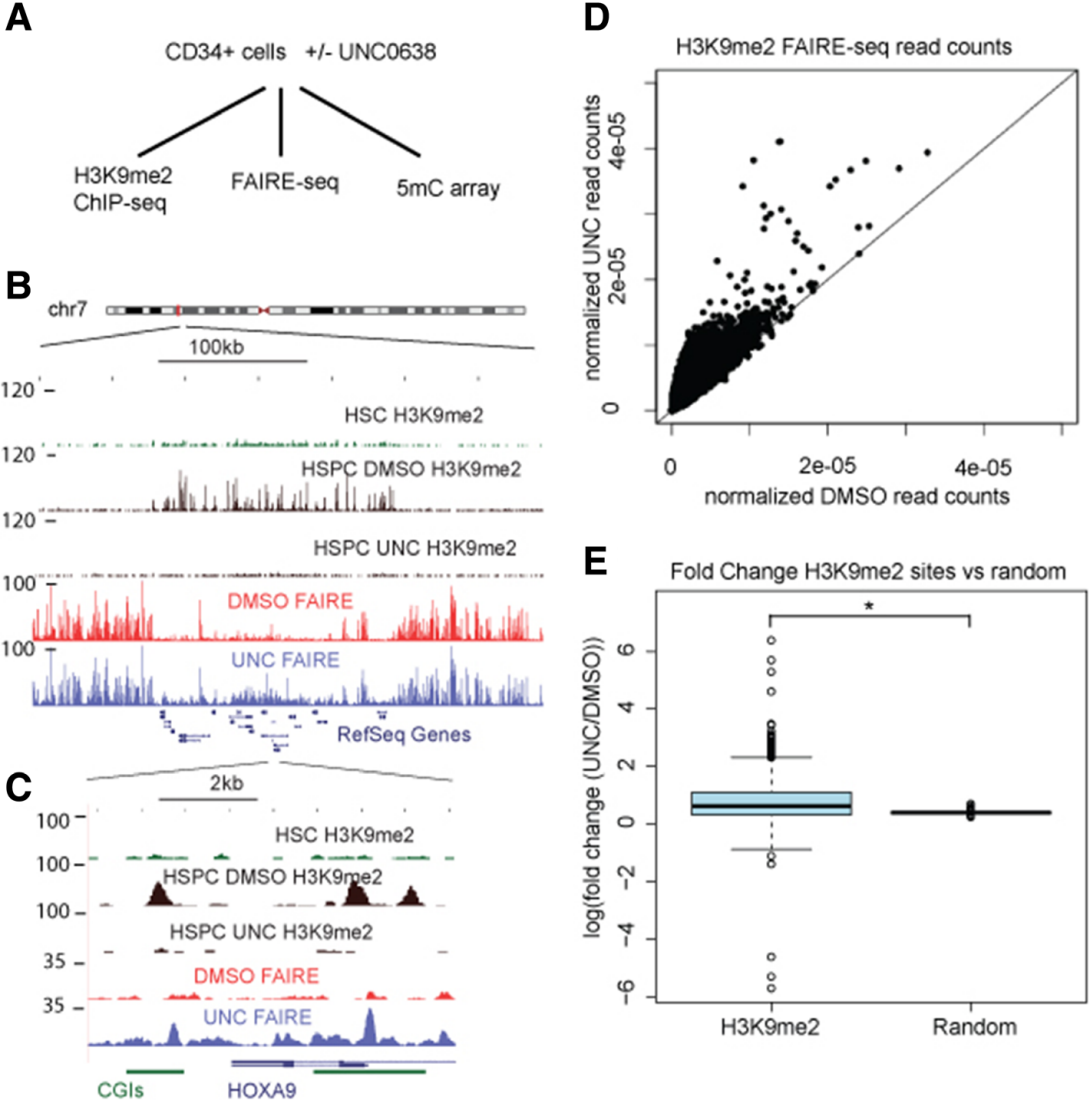
**H3K9me2 is associated with condensed chromatin and removal of H3K9me2 through UNC0638 reverses chromatin condensation. (A)** A flow diagram summarizing the study. See text for details. **(B)** Genome browser screen shot of the HOX locus on chromosome 7 indicating a mutual exclusivity of H3K9me2 and chromatin accessibility as measured with FAIRE as well as an increase in chromatin accessibility upon loss of H3K9me2 following UNC0638 treatment. **(C)** Closer look at the HOXA9 and HOXA10 reveals the relationship between H3K9me2 and chromatin accessibility as measured by FAIRE. **(D)** Scatter plot of normalized FAIRE signals at all H3K9me2 sites in HSPCs for UNC0638- and DMSO-treated samples reveals a general increase of chromatin accessibility at H3K9me2 sites upon UNC0638 treatment. **(E)** Fold change (UNC0638/DMSO) for FAIRE signals at H3K9me2 sites as compared to randomly selected regions. Sites of H3K9me2 nucleation in HSPCs are unique in their response to UNC0638.

Our previous results revealed that H3K9me2 patterning progresses through distinct stages during HSC differentiation. First, H3K9me2 marks appear low or absent in HSC-enriched CD34^+^CD90^+^CD38^lo^CD45RA^–^ cells [6]. Next, in CD34^+^ HSPCs (composed mainly of committed progenitors), a nucleation stage ensues in which H3K9me2 marks appear at discrete loci across the genome [6]. Finally, H3K9me2 marks spread across the genome, presumably in *cis* from sites of nucleation, to form characteristic patterns in mono-lineage cells such as CD41^+^CD61^+^ committed megakaryocytes or CD3^+^ T-cells [6]. For our investigation into the relationship between H3K9me2 patterning and chromatin accessibility, we chose to examine HSPCs given they represent the nucleation stage of H3K9me2 patterning. To do this we performed chromatin accessibility profiling with FAIRE-seq and integrated this data with H3K9me2 ChIP-seq and DNA methylation data from the same cells (Figure 1A). UNC0638 is highly effective at this stage in blocking H3K9me2 nucleation, permitting analysis at sites of nucleation in the absence of H3K9me2 marks. Visual examination of H3K9me2 and FAIRE tracks in CD34^+^ HSPCs revealed mutual exclusivity in signals for H3K9me2 and chromatin accessibility (Figure 1B). Furthermore, closer examination of the genomic profiles indicated that loss of H3K9me2 upon UNC0638 treatment was associated with increase in chromatin accessibility (Figure 1C). This trend was supported by qPCR analysis for FAIRE and H3K9me2 ChIP as well (Additional file 2: Figure S1). These observations are consistent with H3K9me2 leading to chromatin condensation and loss of H3K9me2 leading to an increase in chromatin accessibility.

To examine this on a genome-wide scale, we identified all sites of H3K9me2 nucleation (“peaks”) in CD34^+^ cells and counted the FAIRE reads in DMSO- and UNC0638-treated cells. This analysis revealed increased chromatin accessibility as the predominant behavior at H3K9me2 sites upon treatment with UNC0638 (Figure 1D). To evaluate these results in terms of the background level of chromatin changes across the genome, we randomly sampled sites from the genome and calculated the fold change in FAIRE signal at these randomly chosen regions. Compared to regions of H3K9me2 nucleation, randomly sampled regions had significantly smaller changes (*P* = 2.03577 × 10^-250^; Wilcoxon rank sum test) in chromatin accessibility (Figure 1E). We further evaluated the background changes in chromatin accessibility by sliding 1 kb windows in 50 bp increments across the genome and calculating the fold change of FAIRE read density in UNC0638-treated cells versus control cells. This analysis revealed that changes in FAIRE-seq read densities upon UNC0638 treatment were largely specific to H3K9me2 nucleation sites, indicating that changes in chromatin structure are specific to sites of H3K9me2 nucleation (see Additional file 3: Figure S2).

**Figure 2:**
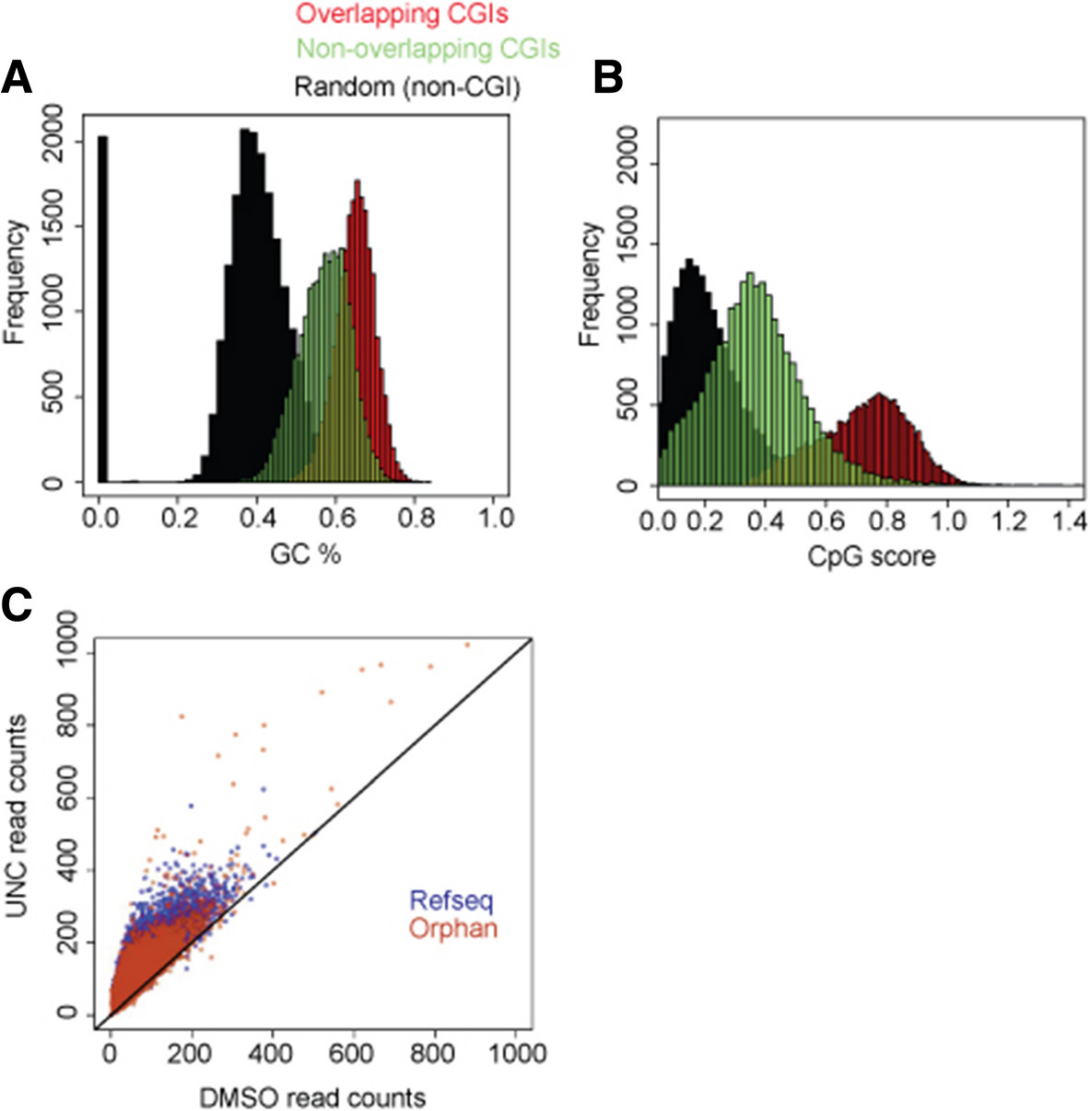
**H3K9me2 is nucleated at CpG-island-like regions across the genome.** Measuring the **(A)** GC% and **(B)** CpG score at H3K9me2 peaks overlapping and non-overlapping CGIs and comparing these to randomly chosen regions indicates that H3K9me2 peaks have GC content similar to CGIs, even if they are not considered “classical” CGIs. **(C)** Scatter plot of FAIRE signals at RefSeq promoter and Orphan CGIs for UNC0638- and DMSO-treated samples. A general increase in chromatin accessibility upon UNC0638 treatment is observed at both Promoter and Orphan CGIs.

Our previous results indicated that a large percentage of the H3K9me2 nucleation sites (~50%) were at CpG islands [6], but it was unclear what the sequence basis of the non-CGI sites was. In order to more extensively investigate the relationship between H3K9me2 peaks, CGIs, and UNC0638-driven changes in chromatin structure, we first reanalyzed overlap with nucleation sites and CGIs as defined by the UCSC Genome Browser [14,15], which classifies 28,691 CGIs throughout the genome. This analysis shows that 48% of nucleation sites overlap CGIs, similar to our previous results. Further examination of the sequence content for both CGI and non-CGI H3K9me2 sites revealed that non-CGI H3K9me2 sites had GC and CpG content, similar to CGIs, but were not meeting the thresholds of classical CGIs (Figure 2A,B) [14].

We next stratified CGIs based on whether they are promoter associated or “orphan” sites, as defined by Illingworth et al. [16] and analyzed UNC0638-driven changes in chromatin. Of the CGIs in the human genome, approximately half are associated with promoters and may play roles in facilitating transcriptional regulation while the other half are found in inter- and intragenic regions and have unknown functions (so-called orphan CGIs) [17]. Analysis of FAIRE read counts across both promoter and orphan CGIs indicates that both sets of CGIs display similar behavior to all sites of H3K9me2 nucleation, with dramatic increases in chromatin accessibility in response to UNC0638 (Figure 2C).

**Figure 3:**
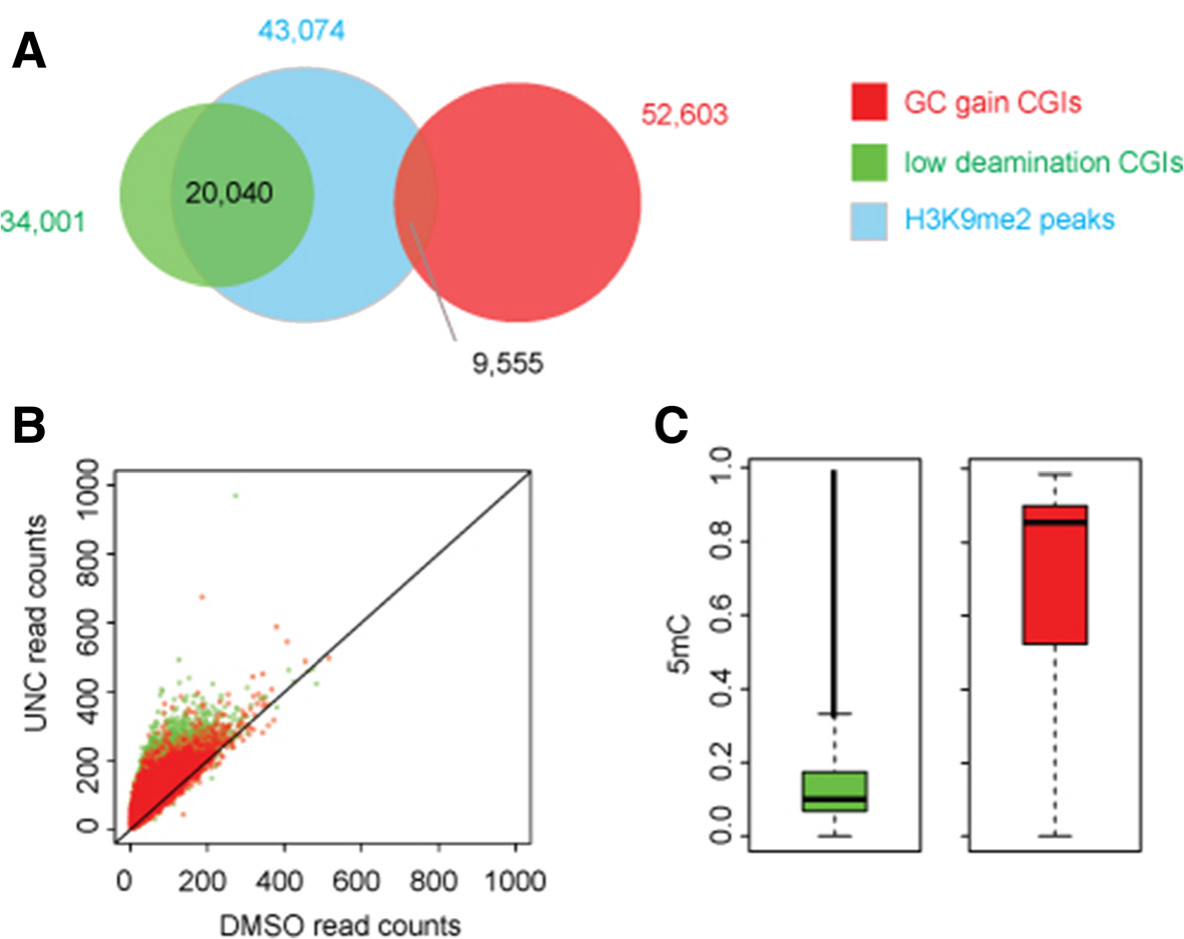
**H3K9me2 nucleation and effect on chromatin accessibility is biased towards the low deamination, mostly unmethylated CGIs. (A)** H3K9me2 nucleation sites (blue) are biased towards CGIs with low rates of C- > T deamination (green) as compared to CGIs that evolutionarily show G/C gain (red). **(B)** The increase in chromatin accessibility is more dramatic at the CGIs with low rates of C- > T deamination (green) as compared to CGIs that evolutionarily have G/C gain (red). **(C)** DNA methylation levels in CD34^+^ HSPCs for CGIs with low rates of C- > T deamination (green) and CGIs displaying G/C gain (red) indicate that the CGIs with low rates of C- > deamination are mostly unmethylated while the GCIs displaying evolutionary gain of G/C content are mostly methylated.

**Figure 4:**
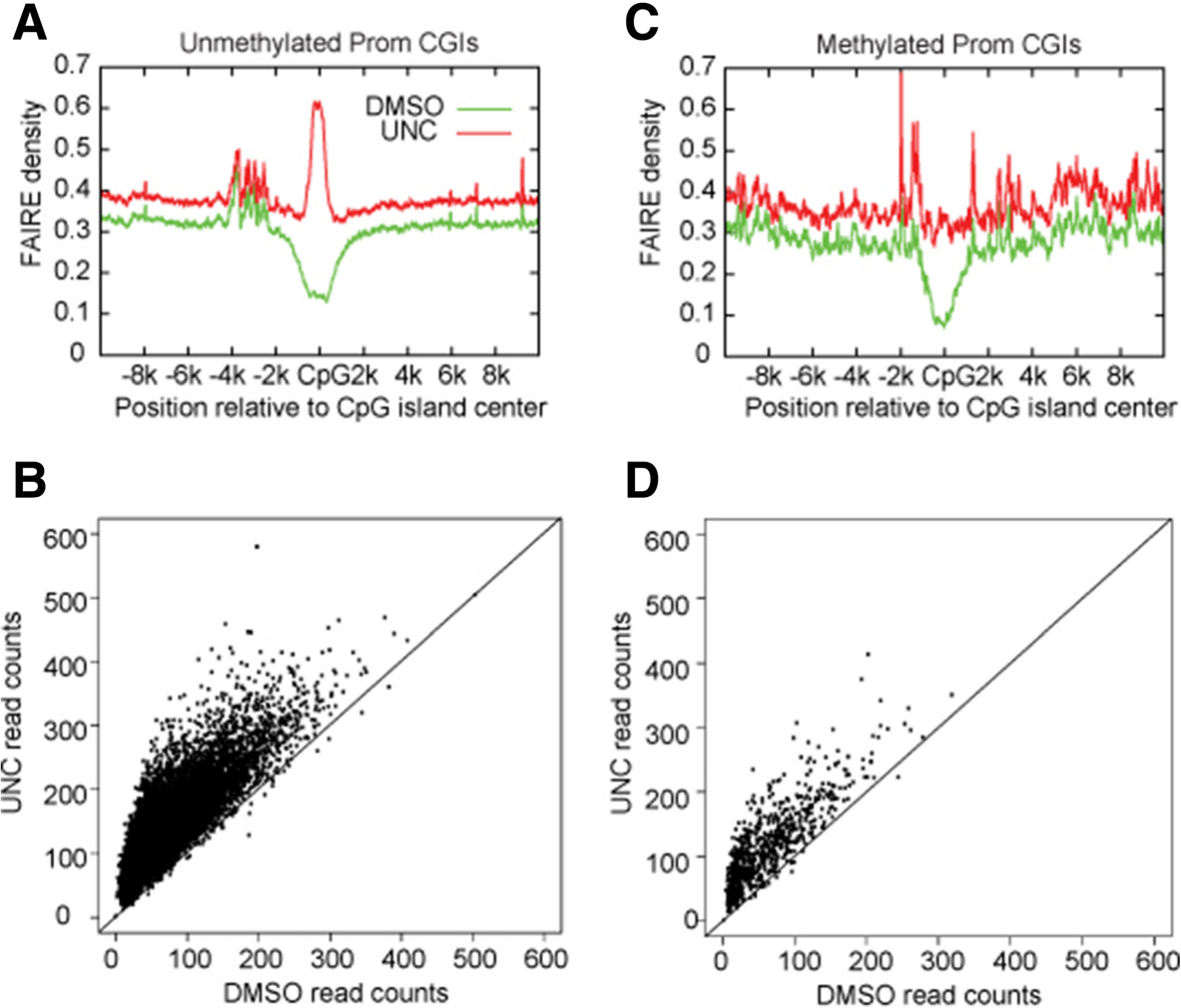
**DNA methylation protects regions of reversal of chromatin condensation upon suppression of H3K9me2. (A)** Aggregate plots of FAIRE density at unmethylated promoter CGIs indicate that chromatin accessibility is completely reversed upon suppression of H3K9me2 for unmethylated promoter CGIs. **(B)** Scatter plot of FAIRE signals at unmethylated promoter CGIs demonstrates that changes in chromatin accessibility under UNC0638 treatment resemble changes at all H3K9me2 sites (Figure 1D). **(C)** Aggregate plots of FAIRE density at methylated promoter CGIs reveals a mitigated increase in chromatin accessibility upon loss of H3K9me2 at methylated promoter CGIs. **(D)** Scatter plot of FAIRE signals at methylated promoter CGIs confirms that methylated CGIs have less change in chromatin accessibility under UNC0638 treatment.

The GC and CpG content of H3K9me2 nucleation sites suggest that H3K9me2 is established in CD34^+^ HSPCs at CpG islands that do not necessarily meet thresholds based on sequence content to be considered CGIs. The initial characterization of CpG islands was based on the experimental observation that there is an enrichment of unmethylated CpG dinucleotides in the mouse genome [18]. Recently, evolutionary analysis of CGIs in primate genomes has been used to classify CGIs in several evolutionary regimes: i) those with low rates of C- > T deamination that are predicted to be mostly unmethylated, ii) those that display rapid G/C gain that are predicted to be constitutively methylated, and iii) those under selection [19]. Examining the overlap of CGIs belonging to these categories with the H3K9me2 nucleation sites revealed that H3K9me2 is preferentially established at CGIs with low rates of C- > T deamination (Figure 3A). We next investigated the effect that loss of H3K9me2 had on chromatin accessibility for CGIs in each group. Each group had an increase in chromatin accessibility upon loss of H3K9me2 (see Additional file 4: Figure S3). For ease of interpretation, we removed CGIs under selection from analysis. Both CGIs with low rates of C- > T deamination and those that rapidly gain G/C content had substantial increases in chromatin accessibility upon loss of H3K9me2, with more dramatic changes for the CGIs with low rates of C- > T deamination (Figure 3B). Examining the DNA methylation status of these regions indicated that, as expected, the CGIs with low rates of C- > T deamination were mostly unmethylated while those CGIs with rapid G/C gain were mostly methylated (Figure 3C).Given these results, we next investigated the potential role of DNA methylation on CGIs in response to UNC0638. We stratified promoter-based CGIs into those that are methylated and those that are unmethylated (Methods) and examined the changes in chromatin accessibility for both groups in response to UNC0638 treatment. The biggest changes occurred at unmethylated CGIs (11,977 total), where UNC0638 dramatically increases chromatin accessibility (Figure 4A,B). In contrast, methylated CGIs (767 total) were more modestly affected, with UNC0638 treatment increasing the FAIRE signal roughly back to background levels (Figure 4C,D). In our analysis, CGIs had low chromatin accessibility regardless of methylation status, consistent with these regions being marked with H3K9me2 in HSPCs. Thus, H3K9me2 patterning is critical for inducing chromatin structure observed at unmethylated CGIs, which represent the majority of CGIs, and to a lesser degree at methylated CGIs.

## Conclusions

In summary, we find that H3K9me2 patterning regulates chromatin structure at promoter and orphan CGIs and other sites of H3K9me2 nucleation, specifically promoting “closed” chromatin states. These results support the notion that G9a/GLP-H3K9me2 participates in global changes in chromatin structure in addition to histone patterning during HSC lineage formation. However, the biological significance of this patterning remains a question. One possibility is that H3K9me2 patterning helps reinforce chromatin states at sites of transcription during lineage specification, which may need to be reset in certain lineages and re-formed *de novo*. To our knowledge, this is the first observation of coordination between H3K9me2 patterning, promoter and orphan CGIs, DNA methylation, and chromatin structure.

## Methods

### Cell culture and treatment

Human CD34^+^ cells from healthy adults were purchased from the Fred Hutchinson Cancer Research Center Cell Processing Shared Resource, as described previously [6]. Unfractionated CD34^+^ cells were treated with 2 μM of UNC0638 or 0.02% DMSO for 48 h, as described previously [6].

### FAIRE-seq

FAIRE was performed as previously described [13]. Paired-end sequencing (100 × 100) was performed in replicate on a HiSeq 2500 to obtain ~50 M reads per replicate. Sequenced reads were aligned to the hg19 build of the human genome (hg19; GRCh37) using bowtie2 [20] with local read alignment. Aligned reads were further filtered to exclude improperly paired reads and duplicate reads. Wiggle tracks were prepared for visualization on the UCSC Genome Browser [15] by sliding 10 bp windows across each chromosome and counting the sequenced fragments overlapping each window; reproducibility of FAIRE tracks was assessed visually and replicate libraries were combined to make final bed files for each condition. Peaks of FAIRE-seq were called with F-seq [21] using default parameters and a 200 bp feature length. Irreproducible Discover Rate analysis [22] was performed to identify reproducible peaks.

### H3K9me2 ChIP-seq

Aligned bam files for H3K9me2 ChIP-seq data were obtained from [6]. Wiggle tracks were generated for visualization on the UCSC Genome Browser [15]. Visual examination of CD34 HSPC H3K9me2 peaks indicated punctate peaks and regions of enrichment were identified using MACS with a *P* value threshold of 1 × 10^-10^. This analysis resulted in 43,159 peaks.

### DNA methylation

DNA methylation results were obtained from [6]. Visualization of DNA methylation levels in CD90^+^ HSCs and CD34^+^ HSPCs (see Additional file 5: Figure S4) revealed a bimodal distribution. CGIs were considered methylated with a methylation score >0.75 and unmethylated with a methylation score <0.25. All regions in between were considered indeterminate.

### H3K9me2 simulation

Simulation of random sites for Figure 2E was performed by randomly choosing 1,000 regions of 1 kb 10,000 times and calculating the fold change of FAIRE signal for UNC0638 over DMSO at each region.

### Data access

The data discussed in this publication have been deposited in NCBI’s Gene Expression Omnibus [23] and are accessible through GEO Series accession number GSE59749.

## Abbreviations

CGI: CpG island; DMSO: Dimethyl sulfoxide; FAIRE: Formaldehyde Assisted Isolation of Regulatory Elements; HSC: Hematopoietic stem cell; HSPCs: Hematopoietic stem and progenitor cells; H3K9me2: Histone H3 Lysine 9 di-methylation.

## Competing interests

The authors declare that they have no competing interests.

## Authors' contributions

DES and PJP designed the study. XC, CT, and RS carried out experiments and analysis. All authors discussed the results. DES and PJP wrote the manuscript. All authors read and approved the final manuscript.

## Supplementary Material

Additional file 1: Table S1 FAIRE-seq statistics for UNC0638- and DMSO-treated cells. Click here for file

Additional file 2: Figure S1 qPCR validation of FAIRE-seq and ChIP-seq results. H3K9me2 ChIP-seq and FARE-seq profiles for DMSO- and UNC0638-treated cells at the (A) HOXA9 and (B) MLLT11 loci. Primer coordinates for qPCR are indicated by green bars. (C) Corresponding qPCR results. Relative values represent qPCR values normalized to GAPDH. Click here for file

Additional file 3: Figure S2 FAIRE fold change for H3K9me2 nucleation sites as compared to the whole genome. The increase in chromatin accessibility as measured by the log of the fold change of the FAIRE signal for UNC0638 over DMSO for all H3K9me2 nucleation sites (H3K9me2) as compared to the log of the fold change for FAIRE (UNC0638/DMSO) for all 1 kb windows across the genome tiled in 50 bp increments (Background). Click here for file

Additional file 4: Figure S3 The FAIRE signals for UNC0638 and DMSO for three classes of CGIs: (green) those with low rates of C- > T deamination that are predicted to be mostly unmethylated, (red) those that display rapid G/C gain that are predicted to be constitutively methylated, and (blue) those under selection. Click here for file

Additional file 5: Figure S4 DNA methylation distributions for probes in (A) CD34^+^ DMSO and (B) CD90^+^ cells. Click here for file
