# Supplementary material for: G9a/GLP-dependent H3K9me2 patterning alters chromatin structure at CpG islands in hematopoietic progenitors
Source: Epigenetics Chromatin. 2014 Sep 10;7:23. doi: 10.1186/1756-8935-7-23 (PMC4166411; doi:10.1186/1756-8935-7-23)
Supplement: Additional file 2: Figure S1 — qPCR validation of FAIRE-seq and ChIP-seq results. H3K9me2 ChIP-seq and FARE-seq profiles for DMSO- and UNC0638-treated cells at the (A) HOXA9 and (B) MLLT11 loci. Primer coordinates for qPCR are indicated by green bars. (C) Corresponding qPCR results. Relative values represent qPCR values normalized to GAPDH. [file 1756-8935-7-23-S2.pdf]

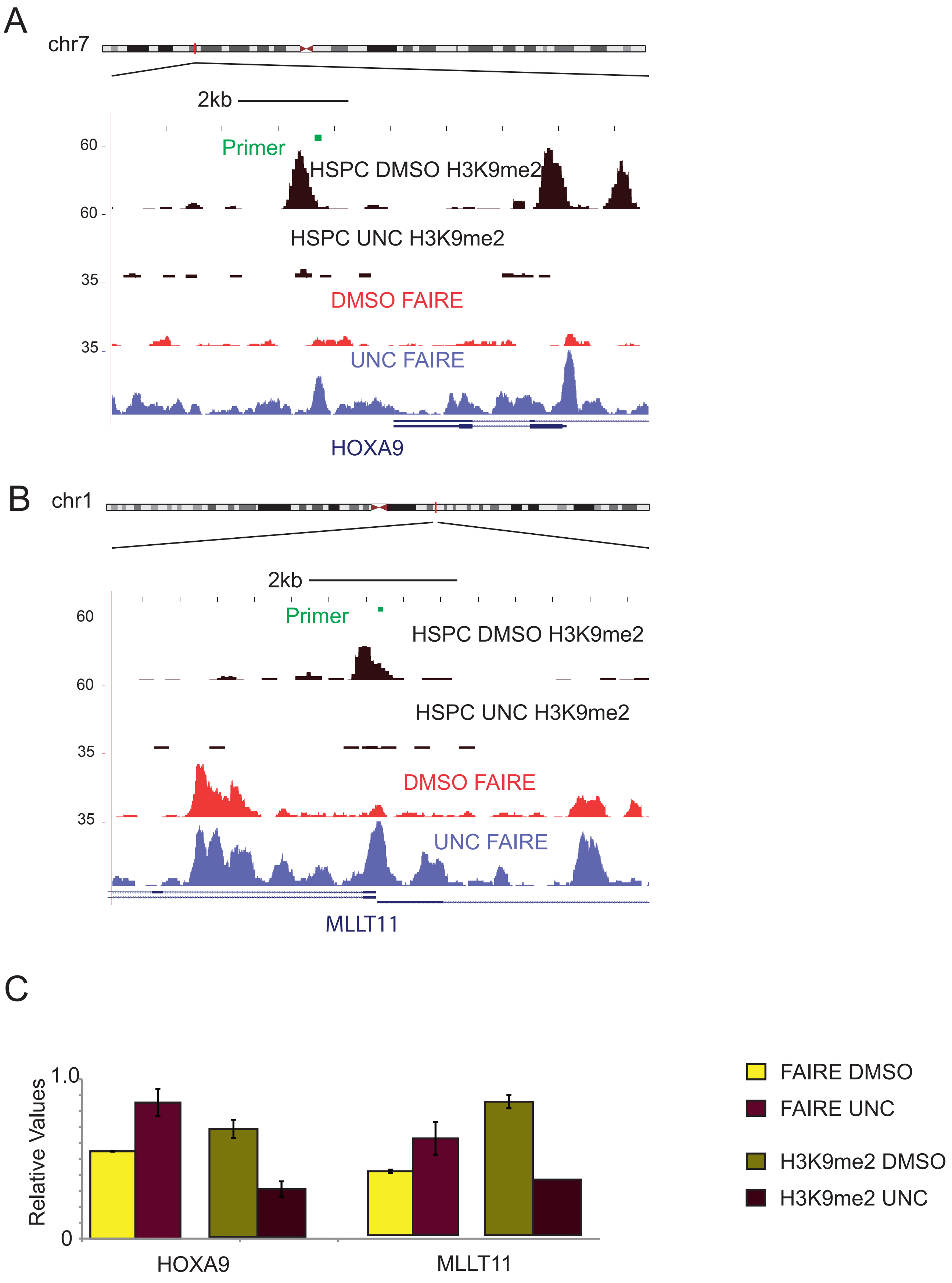

Figure S1 - qPCR validation of FAIRE-seq and ChIP-seq results. H3K9me2 ChIP-seq and FARE-seq profiles for DMSO and UNC0638 treated cells at the (A) HOXA9 and (B) MLLT11 loci. Primer coordinates for qPCR are indicated by green bars. (C) Corresponding qPCR results. Relative values represent qPCR values normalized to GAPDH.
