# Supplementary material for: G9a/GLP-dependent H3K9me2 patterning alters chromatin structure at CpG islands in hematopoietic progenitors
Source: Epigenetics Chromatin. 2014 Sep 10;7:23. doi: 10.1186/1756-8935-7-23 (PMC4166411; doi:10.1186/1756-8935-7-23)
Supplement: Additional file 3: Figure S2 — FAIRE fold change for H3K9me2 nucleation sites as compared to the whole genome. The increase in chromatin accessibility as measured by the log of the fold change of the FAIRE signal for UNC0638 over DMSO for all H3K9me2 nucleation sites (H3K9me2) as compared to the log of the fold change for FAIRE (UNC0638/DMSO) for all 1 kb windows across the genome tiled in 50 bp increments (Background). [file 1756-8935-7-23-S3.pdf]

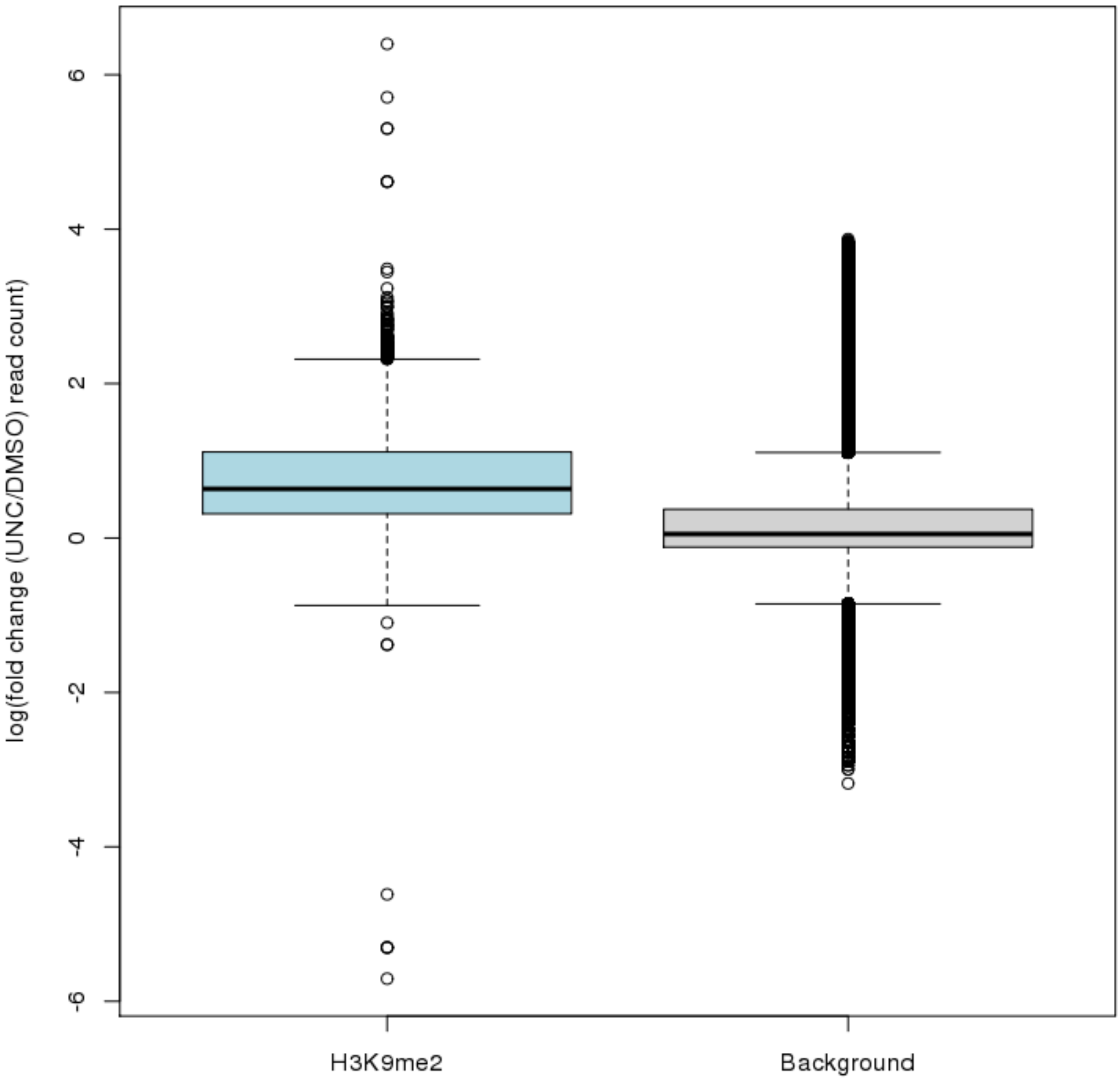

Figure S2 – FAIRE fold change for H3K9me2 nucleation sites as compared to the whole genome.

The increase in chromatin accessibility as measured by the log of the fold change of the FAIRE signal for UNC0638 over DMSO for all H3K9me2 nucleation sites (H3K9me2) as compared to the log of the fold change for FAIRE (UNC0638/DMSO) for all 1kb windows across the genome tiled in 50bp increments (Background).
