# Supplementary material for: G9a/GLP-dependent H3K9me2 patterning alters chromatin structure at CpG islands in hematopoietic progenitors
Source: Epigenetics Chromatin. 2014 Sep 10;7:23. doi: 10.1186/1756-8935-7-23 (PMC4166411; doi:10.1186/1756-8935-7-23)
Supplement: Additional file 4: Figure S3 — The FAIRE signals for UNC0638 and DMSO for three classes of CGIs: (green) those with low rates of C- > T deamination that are predicted to be mostly unmethylated, (red) those that display rapid G/C gain that are predicted to be constitutively methylated, and (blue) those under selection. [file 1756-8935-7-23-S4.pdf]

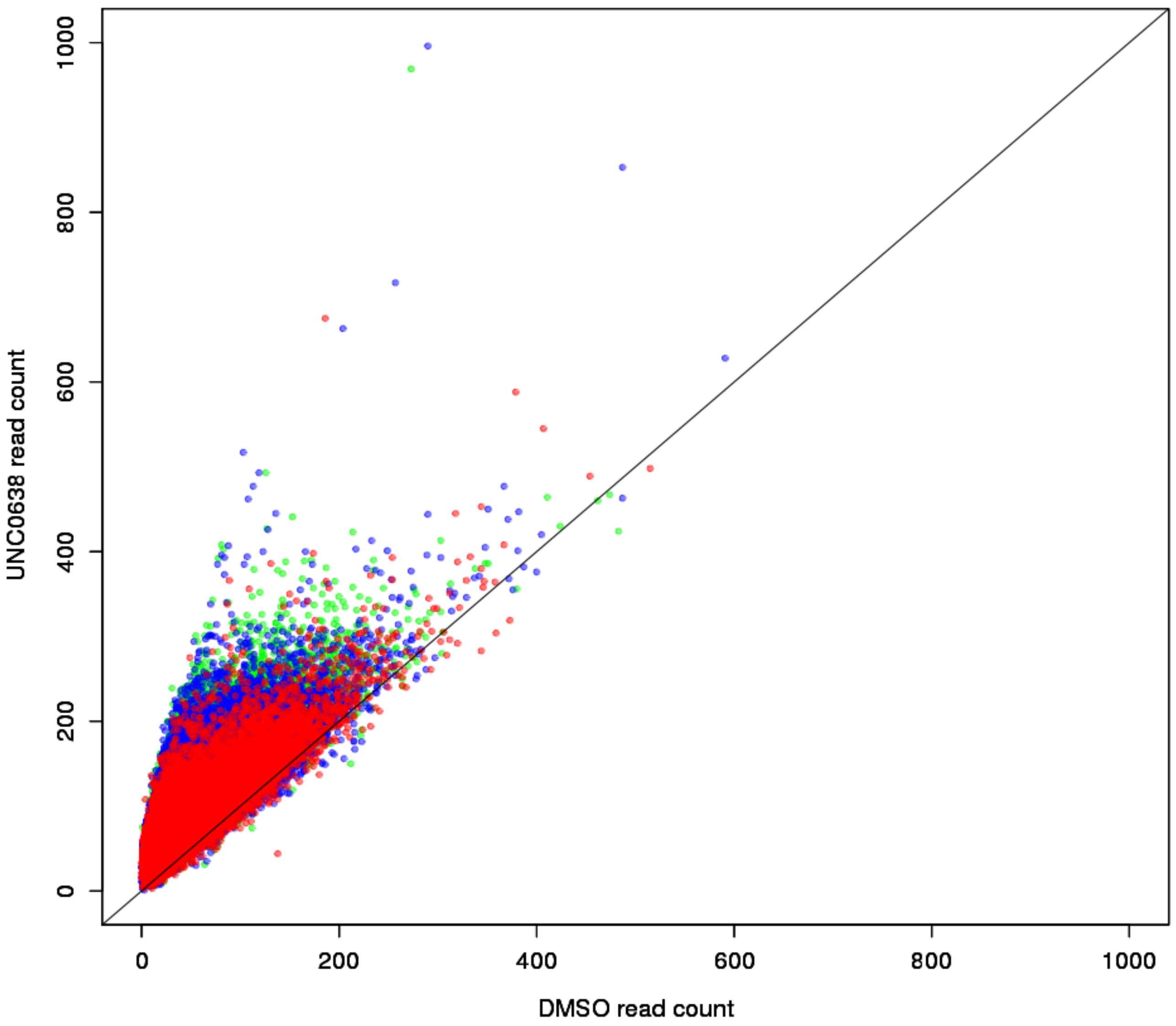


**Figure S3 – The FAIRE signals for UNC0638 and DMSO for three classes of CGIs**: (green) those with low rates of C->T deamination that are predicted to be mostly unmethylated, (red) those that display rapid G/C gain that are predicted to be constitutively methylated and (blue) those under selection.
