# Supplementary figures and images for: G9a/GLP-dependent H3K9me2 patterning alters chromatin structure at CpG islands in hematopoietic progenitors
Source: Epigenetics Chromatin. 2014 Sep 10;7:23. doi: 10.1186/1756-8935-7-23 (PMC4166411; doi:10.1186/1756-8935-7-23)

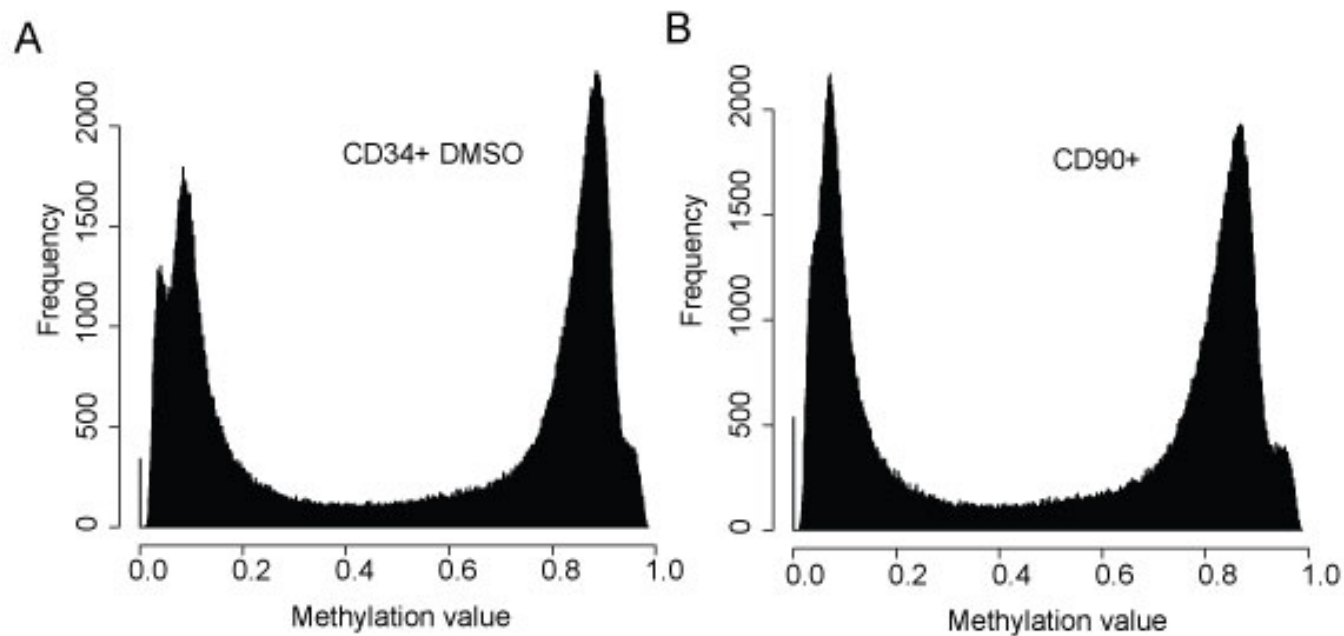

**Figure S4 – DNA methylation distributions for probes in (A) CD34+ DMSO and (B) CD90+ cells.**

Supplement: Additional file 5: Figure S4 — DNA methylation distributions for probes in (A) CD34+ DMSO and (B) CD90+ cells. [file 1756-8935-7-23-S5.pdf]
